# The future of polarisation in Europe: relative cosmopolitanism and democracy

**DOI:** 10.1186/s40309-021-00183-2

**Published:** 2021-10-10

**Authors:** Markus Pausch

**Affiliations:** Salzburg University of Applied Sciences, Puch bei Salzburg, Austria

**Keywords:** Democracy, Polarisation, Cosmopolitanism, Communitarianism, Europe, European Union

## Abstract

One of the central features of our societies is an increasing polarisation between communitarian and cosmopolitan positions. The theoretically sound and differentiated concepts are increasingly being escalated and misused in political practice by authoritarian populists and polarising pushers who try to pull the undecided to their side and tear society apart. Two essential agreements of the post-war period are increasingly being called into question: The European consensus, which considers European unification as an essential achievement and goal of political actors, and the democratic consensus, which states that representative democracy is the undisputed best form of government. In this article, after an introductory definition of polarisation, two future scenarios are developed. In the scenario “Polarised Europe”, polarisation is extrapolated into the future and discussed with its serious consequences for the democratic and European consensus. The second scenario “Democratised Europe” shows how the concept of a relative cosmopolitanism can mitigate polarisation and what steps could possibly be taken to constructively turn it into a more democratic direction.

## Introduction: The end of the European and the democratic consensus

The history of most Western European states after 1945 and most central or Eastern European states after 1989 is characterised by two main agreements of its elites and citizens: a democratic consensus and a European consensus [3, 29, 31, 35]. The democratic consensus refers to the broad endorsement of representative democracy, the European consensus to the broad endorsement of the European integration process. This does not mean that there have not been voices against democracy and Europe on the fringes of the political spectrum. Nor does it mean that democracy and European unification must be necessarily seen as an interdependent pair. It merely means that until the 1990s no broad public or major party seriously questioned one or the other. The concrete forms of the nation-state democracies remained just as unaffected by this consensus as the concrete forms of the European Communities and later the European Union. For a long time, these two agreements were strong enough to cover up the smouldering lines of conflict underneath. In the crises of recent years, which many consider existential, they are now erupting and coming to a head in a widely visible polarisation [22]. The ideal images of what Europe and democracy are supposed to be differ gravely and differentiate towards the poles of the conceivable spectrum. This is not to say that democratic societies must be based primarily on consensus-building. Conflict is intrinsic to democracies. Nevertheless, it is important to reach agreement on fundamental issues and to find mechanisms of conflict resolution that do not erode the political system [6]. For the EU, it can be critically noted that conflicts have been covered up by elite consensus for too long without sufficient democratic debate in a European public sphere [10].

The main cleavage today is both on the question of Europe and on the question of democracy, between cosmopolitan currents on the one hand and communitarian ones on the other. In this article, the dissolution of the two consensuses is thought further into the future and scenarios are presented to show what consequences are possible. After introductory theoretical considerations on the connection between polarisation and democracy and the cleavage between cosmopolitanism and communitarianism, two scenarios are presented: In scenario 1 “Polarised Europe”, polarisation leads to the erosion of democracy and the European integration process. In scenario 2, “Democratised Europe”, the contradictions between communitarian and cosmopolitan ideas are dialectically resolved with a concept of relative cosmopolitanism, structural reforms and concrete democratic innovations.

## Polarisation between communitarian and cosmopolitan positions

In political science, polarisation refers to a state or process of hardened differences of opinion that are based on perceived or actual inequalities ([15], 693). In pluralistic societies, conflicts are immanent. The purpose of democracies is not to cover up existing conflicts, but to discuss them constructively and transparently. In this respect, a certain degree of polarisation is not only normal but necessary in democracies. However, the challenge is to prevent polarised conflicts from sliding into violent confrontation where dialogue comes at its end. There are a number of theories that explain political polarisation processes. The theory of pluralism assumes that different worldviews and political interests are normatively legitimate and are to be negotiated in a model of democratic governance. To this end, Richard Bellamy has described four constitutional forms, which in turn are based on different political theory models [6]. Constitutional neutrality means that compromise is achieved by trimming and that the constitution is neutral towards world views. “Contentious opinions can still be expressed, but only in private arenas such as pubs and clubs or with friends and family” ([6], 202). “Interest Group Pluralism or compromise by trading” is about the balancing of interests between relevant social and political groups. “…[W]hen conflicts occur, groups will always be able to force and reach mutually beneficial trade-offs with others” (ibid 2000, 204). We know this from collective bargaining between workers and employers in a social partnership context. In the model “Compromise by Segregation”, power sharing among different groups is considered to be the best approach. It is all about the rights of groups and the group’s control of questions in their vital interests. “They seek to preserve a group’s control of as many areas vital to its form of life as possible, to protect other aspects against damaging incursions and to ensure the necessary collective decisions are consensual.” (ibid. 2000, 106 f.). The problem with this model is that it can lead to a hardening of group identity and thus to polarisation. That is why the fourth constitutional model described by Bellamy—“Compromise as Negotiation”—seems to be more suitable for the mitigation of polarisation than consociationalism since it acknowledges pluralism and focusses on dialogue. “The key disposition to foster is encapsulated in the republican formula ‘audi alteram partem’ or ‘hear the other side’. This criterion constrains both the procedures and the outcomes of the political process. People must drop purely self-referential or self-interested reasoning and look for considerations others can find compelling, thereby ruling out arguments that fail to treat all of equal moral worth. They must strive to accommodate the clashes of preferences and principles associated with pluralism by seeking integrative compromises that view the concerns raised by others as matters to be met rather than constraints to be overcome through minimal, tactical concessions” (ibid. 2000, 211 f.). This negotiation-based approach fits with a theory of democracy based on dialogue, deliberation and participation which in recent decades has been strongly associated with Jürgen Habermas [26]. Although Bellamy and Castiglione have discussed and criticised Habermas’ theory of constitutional patriotism elsewhere [7], they share the general idea that democracy must be based on dialogue and the inclusion of the other [25]. Later in this article, elements of Habermas’ discourse theory are taken up again.

In any case, dealing with conflict is essentially one of the central questions of democratic theory. This seems particularly true for the twenty-first century [43]. And this this dealing with conflicts depends on normative positions, which are themselves subject of polarisation. One aspect of social division is the exaggeration or abridgement of theoretical concepts and often their deliberate misuse. We have been experiencing this for some years around the concepts of communitarianism and cosmopolitanism. Both stem from a multifaceted philosophical debate. Bellamy and Castiglione argue that they are ontological rather than ideological ([7], 187). However, in times of polarisation, they are misused by political parties as absolute truths and models of exclusion.

Communitarian approaches assume a common cultural core that holds societies together and that should not be disturbed too much [18, 60, 61]. For a long time, this idea was advocated by thinkers of the left who used it to defend welfare state achievements against neoliberal globalisation. For some years now, nationalists have been using the core augmentation of communitarianism without calling it by its name. They plead for a retreat to the national and thus undermine the philosophical concept with their racist, ethnicist and authoritarian world views [41].

Cosmopolitan assumptions assume the possibility of universal democracy and a negotiation process that can be described with Juergen Habermas [24] as procedural rationality which does not presuppose any cultural preconditions. The philosophical and political theoretical definitions of cosmopolitanism [2, 4, 44] are almost always based on an inclusive conception of the world, universalist ideas in which all people are granted equal rights. In recent years, cosmopolitanism is also closely linked to ecological questions [13]. But despite the claim of global solidarity and equality among political thinkers of cosmopolitanism, political actors who represent cosmopolitan ideas in the political party spectre or in civil society and NGOs sometimes ignore socio-economic conditions of poorer milieus and underestimate achievements of nation state democracy [41]. This can drive polarisation and de facto exclude certain groups from political discourse [45].

In terms of political theory, one could argue that the two schools of thought serve different human needs: communitarians prioritise belonging to a nation or a political entity, while cosmopolitans prioritise equal freedom for all people [20, 48]. In this article, the distinction does not stand for all political-theoretical facets of the two schools of thought. Rather, they describe the misuse in the political debate that is based on fundamental orientations and worldviews. That is why I will use the terms exclusive communitarianism and dogmatic cosmopolitanism to avoid confusing the practical aberrations with the differentiated theoretical concepts.

For Wolfgang Merkel, this new line of conflict is emerging as a growing cleavage between winners and losers of globalisation. While the former are mostly well educated and wealthy, the latter suffer from the risks of globalisation because they have less capital and resources and are less represented in the political system. For Merkel, this gap of representation is exploited by the far right and drives polarisation ([41], 9). But some dogmatic cosmopolitans in the public debate also contribute to polarisation by discrediting others, by politicising lifestyle issues offensively and sometimes without regard to socio-economic conditions of poorer milieus ([52], Sandel). They underestimate the achievements of the nation states in terms of democratic quality and think that democracy is easily transferable to a supranational or even global level (Merkel). People who are formally less educated, less wealthy and who are threatened by globalisation effects sometimes perceive the politicisation of life styles as a kind of cosmopolitan arrogance [5] or lifestyle arrogance [55]. The polarisation is based on a factual and increasing inequality that has been observed for a long time and has been empirically documented in many ways [27, 50, 58].

In this article, I draw on Merkel’s argumentation that globalisation is a key factor for polarisation between cosmopolitan and communitarian positions on democracy and Europe. But I assume that it is not only a question of winners and losers of globalisation that determines whether people tend to lean one way or the other. Rather, in a dialectical understanding of the world, different needs are opposed to each other, which are ideally represented by the two [20]. Communitarians tend to serve the need for belonging and security, while cosmopolitanism is more oriented towards individual freedom and universal solidarity.

With reference to the theories of democracy as rebellion [48] and a relative or rebellious cosmopolitanism [28], I assume that individuals and societies are torn between two needs: that of freedom and that of belonging. Polarisation processes reflect this existential contradiction, as can be seen in the struggle between communitarianism and cosmopolitanism. If the balance between the two needs falters in a political system, which can happen quickly due to crises and a lack of political equality, polarisation occurs. The dialectical resolution of the conflict is offered by the theoretical concept of relative or rebellious cosmopolitanism that can build bridges between communitarians and cosmopolitans and foster political equality and by the practical strengthening of democracy and dialogue.

As I argued elsewhere, democracy as rebellion means that the individual rebellion against authoritarianism and inequality is rooted in an immediate existential experience and expresses itself as universal solidarity between strangers [12, 48]. This cosmopolitanism is not based on the hybris of being a winner. It is not a theoretical or economic cosmopolitanism, but a rooted cosmopolitanism [4, 59]. Patrick Hayden defined what he calls “rebellious cosmopolitanism” as follows: “…the idea of cosmopolitanism should be situated in a post-foundationalist and post-teleological nexus to prevent it becoming a new political ideology of immutable truth… cosmopolitanism must strive against the injustices of a deeply divided world, yet at the same time accept theoretical, factual, and moral limits on its vision and actions.” ([28], 194). I suggest to call this kind of cosmopolitanism relative cosmopolitanism, in the sense of a relative utopia, as Albert Camus understood it ([47], 52).

The political polarisation that we observe in recent years, is linked to various factors of political equality [11], including representation, participation, transparency, but is also a dialectical relationship between contradictory needs for freedom and belonging, to which cosmopolitanism and communitarianism correspond at the political level. A balance between these needs requires a non-dogmatic relative cosmopolitanism that is based on real life experiences and competences of democracy, dialogue and citizenship education.

### Basis characteristics of polarisation

In a complex world where there are no simple explanations, the risk of polarisation increases. Unequal power relations, socio-economic inequality, structural marginalisation, discrimination or exclusion of certain groups can drive its pernicious forms. The polarisation we encounter today thus has an existential basis, the contradiction between the need for freedom and that for belonging.

Four features characterise polarisation processes [49].

* Discrepancy of opinions: Two clearly identifiable and profiled opinions oppose each other. These opinions are not compatible and configure themselves in an either/or relationship. The communitarians aim at a narrower concept of belonging, the cosmopolitans at a broad understanding in which individual freedom and solidarity are thought globally and universally.

* Group formation: The two opinions are held by two different groups whose members are aware of the discrepancy and feel they belong to one of the two groups. The world is divided into “Us versus Them” [9]. Political opponents are increasingly becoming antagonists [36] or even enemies. Carl Schmitt’s friend-enemy scheme has recently received greater attention again [40, 56]. In political science, the term “affective polarisation” refers to the mutual dislike of the groups (cf. [33]). What is necessary is the awareness that one’s own opinion is one pole in a spectrum that can contain many opinions and that one’s position is represented by a group that is visible in some way. Often these groups give themselves a name, or names are attributed to them. With regard to positioning on the EU, we know the attributions as pro- and anti-Europeans or Eurosceptics. With regard to positioning on democracy, it is mainly a distinction between representative and direct democratic elements.

* Purism: Relative positions are not considered by the two groups. A conciliatory position is rejected. The groups that form the poles in a polarisation process cannot take a middle position because their opinions are too far apart. A drastic example can illustrate this: opponents of the death penalty cannot negotiate about the death penalty. Their position is non-negotiable. The same is true for human rights activists. The historical fighters for democracy could not negotiate their goal with those who wanted to preserve their authoritarian power. Someone fighting for women’s rights cannot soften or weaken the goal of equality. Conversely, authoritarian forces that oppose emancipation do not give an inch. The positions at the poles are therefore fundamentally non-negotiable for the representatives of these poles. This is also true in the case of the polarisation around Europe. Those who advocate a European republic will not discuss the possibility of renationalisation. The reverse is also true.

* Political struggle: The fourth characteristic to be mentioned is that a political struggle for positions must be waged in order to speak of polarisation. The mere existence of major differences of opinion is not per se politically relevant, because it would also be conceivable that one of the groups or even both simply exist in silence without engaging in a political struggle. Only when there is a dispute in public can we talk about polarisation.

However, polarisation processes are not to be regarded as dangerous or endangering democracy per se. To a certain extent, they are part of pluralistic societies. Historically, polarisation processes have even often been a precondition for social change towards more democracy. Polarisation often starts from below and develops bottom-up. When social movements recognise a lack of justice or opportunities for themselves or other groups and fight against it, a hardening of positions is to be expected at first, as the dominant or privileged groups feel threatened and may reject the demands. Only when the pressure of the social movement becomes so strong that it leads to a concession can polarisation develop towards democratisation. For this to happen, the polarisation process must be turned around positively through dialogue and inclusion (cf. [39], 234).

In contrast, a more dangerous form of polarisation develops as an ever sharper intensification of positions in the broad social centre, which can ultimately lead to a willingness to use violence. In such a case, the political differences of opinion lead to strong distrust and hardening and spread relatively quickly among the supporters of the respective side, far into the centre of society. Polarisation leads to social division and the end of dialogue. It is often driven and deliberately fuelled by populist politicians. “Whoever is not for me is against me” is the pointed formula that describes this phenomenon. The use of media plays an important role in this. Media power is therefore of utmost importance in polarisation processes. Communication methods that contradict the dialogue principle and are based on monologues are typically used by divisive politicians.

Two paths can be taken in polarisation processes. The increase in polarisation can lead to a hardening of positions and tear the centre apart. The consequence is that dialogue ends, the desire to destroy and eliminate opponents grows and ultimately violence is used. This process is called pernicious or undemocratic polarisation. The other way polarisation can go is the constructive turn, in which the division is resolved through dialogue. This can be called benign or democratic polarisation [49]. The progression of polarisation depends on the development of structural, socio-economic and political inequalities, on experiences of democracy and on offers of dialogue. The actors of polarisation are divided into pushers and followers who have an interest in division, seek scapegoats and use manipulative communication techniques. In contrast, the so-called bridge builders can counteract this by promoting democracy and dialogue at various political levels [9].

### Polarisation and the end of the democratic consensus

The democratic consensus of the post-war period was based on the conviction that democracy was the only form of government that is acceptable. This consensus was upheld in the political party landscape of Western Europe and, after 1989, largely in Eastern Europe as well. Although there were very different ideas and forms of democratic systems in detail, from a semi-presidential French democracy to a parliamentary form in many other states and further gradations, the basic idea of representative democracy was not in question. This changed in the 1990s and finally reached its peak in the last years. Although there is still a very high percentage of people favouring democracy over all other forms of government in opinion surveys, there is a lot of evidence for a consequent, subversive decline of approval for democracy if we look at other indicators. Not only is the number of parties questioning the democratic consensus increasingly [54], but the desire for a strong leader who does not need to care about parliament or election is also growing [29]. Satisfaction with, trust in and approval of representative democracy and its actors are also declining. Thus, all these factors are eroding the democratic consensus. This mood, a rising inequality and a general uncertainty related to the phenomena of individualisation, but also to concrete, existential experiences of crisis, promote politicisation and polarisation [41]. Authoritarian populists, pushers of polarisation use this for their own goals. With manipulative communication, hate speech and the spreading of fake news [1], they try to further divide society.

Trust in representative democracy is declining not only among losers of globalisation, but among different groups. For exclusive communitarians and nationalists, it is losing its core and dissolving too much in a global world with phenomena like immigration and a questioning of identity; for the other side, it is still too strongly tied to cultural and national identities and structures to develop beyond the nation state. The proposed solutions are as different as the starting points. Exclusive communitarian positions aim at a return to national, sometimes even regional sovereignty. Cosmopolitan positions call for the overcoming of national states, for supranational and global forms of democracy.

### Polarisation and the end of the European consensus

The second basic consensus of the post-war period, which has been wavering in Europe for several decades now, is the European consensus. Although there were always some opponents to the European integration project, they were largely marginal until well into the 1990s [31]. That there was a consensus on the basic idea of unification despite all the differences between intergovernmental and federal conceptions of Europe can be shown by the positions of the governing parties, at least in continental Europe, throughout the second half of the twentieth century [38]. From a democratic perspective, this broad elite consensus, also known as permissive consensus, has always been a problem for it was accompanied by the absence of democratic negotiation processes in a European public sphere [30, 46].

Only with the entry of the FPÖ into a government in Austria in 2000 was this tradition broken, when for the first time an openly anti-European or at least very Eurosceptic party assumed government responsibility. The reactions from other EU countries were very negative at the time. In particular, French President Chirac and German Chancellor Schröder criticised the Austrian People’s Party for entering into a coalition with the right-wing populists. These two politicians not only represented the leading countries in the EU, but also the two big party families, conservatives and social democrats who at that time were already under pressure by the rise of the far right [19].

Today, we know that this was only the beginning of a general European development in which Eurosceptic to anti-European parties came to power in many countries. Italy, the Netherlands, Finland, Belgium and others have since had similar coalitions with right-wing populist or far-right participation. In Hungary and Poland, governments have been established that question basic values of the Union and democracy. The British even decided to leave the EU. All this underlines the fact that the post-war European consensus no longer holds up unchallenged. Europe and its future have become the subject of polarised debates. Again, communitarian and cosmopolitan positions confront each other. Communitarians aim for a return to the nation state. They argue for the dissolution of the EU or the withdrawal of their countries, or at least for renationalisation to restore the full sovereignty of all member states. This would be tantamount to an end of the Union. The cosmopolitans, on the other hand, see the EU as the first step towards overcoming national egoisms and want a federal union that is committed to cosmopolitan values both internally and on the international stage [17]

## Democracy and Europe: two scenarios

In the following, two possible scenarios are considered in a descriptive manner and on the basis of a few indicators. These are narrative scenarios that are intended to describe plausible developments in a qualitative way and to show what would be expected in the event of changes in individual indicators. In doing so, a rough structure is used and the complexity of reality is greatly reduced. The aim of this approach is to highlight orientation points for future developments and the significance of the chosen individual indicators. The indicators chosen for the narrative scenarios relate to two interrelated goals and principles of democracy: political equality and attitudes towards democracy. These in turn affect the principles of freedom and control as described in the Democracy Barometer [11]. The scenarios describe how the polarisation between communitarian and cosmopolitan positions would develop based on the indicators of political equality and democratic attitudes. In the first (Scenario 1: Polarised Europe), there is a further polarisation between exclusive communitarian and dogmatic cosmopolitan ideas. The second scenario “Democratised Europe” shows how the concept of rebellious cosmopolitanism could be used to achieve a mediating and democracy-promoting alternative.

### Scenario 1: Polarised Europe—exclusive communitarianism and dogmatic cosmopolitanism

In the first scenario “Polarised Europe”, I assume that in the polarisation process the nationalist arguments become increasingly strong. The battle for the undecided voters is heating up. The main social and political problems like structural inequality and a lack of democratic experiences remain and help polarising pushers to mobilise followers and find scapegoats among immigrants and political elites. In response to this exclusive communitarian, nationalist aggression, other political actors do not succeed or are not interested in providing more equal chances, participation and representation. They do not seek ways to build bridges to the undecided, but polarise themselves, strongly politicise all nuances of lifestyles and show a certain arrogance as described by Sandel [55] or Beckstein [5]. This leads to parts of the hitherto silent centre choosing one of the two sides and considering the respective opponents as enemies. This is all driven by deficits in the quality of democracy in the nation state, but also at the supranational level.

The four criteria of polarisation are clearly evident. Opinions between dogmatic cosmopolitan and exclusive communitarian positions continue to diverge, fuelled by European policy issues, migration and exclusion of certain groups demanding their rights. “Us versus them” thinking is intensifying, the groups are profiling themselves. Right-wing nationalist parties are at one end. Greens and liberals at the other. Social democracy and conservatives are also marked by conflicts around these issues.

The representation of underprivileged groups, the so-called globalisation losers, in the political process and in the party landscape is low and even declining. The impression that democracy is an elite process is hardening. Cases of corruption among politicians weaken trust in the political parties. The COVID-19 crisis and other existential threats deepen inequality and foster a feeling of insecurity in those groups of the population who are underprivileged or who are at risk of unemployment. But also people in secure economic circumstances who nevertheless have only little experience of democracy in their lives and are used to authoritarian conditions in their families, workplaces and socialisation, show a low degree of willingness to engage in dialogue and tend more and more to criticise democracy. In a world full of crises, the two old agreements, the European consensus and the democratic consensus, seem to be outdated and inefficient. Democracies on the national level often lack representation, transparency and equal participation. Neoliberal economic conditions exploit people and do not allow for democratic experiences and self-efficacy in daily lives.

This situation is exploited by authoritarian populists and polarising pushers who not only stir up opposition to European unification, but also to representative democracy. In their efforts to divide society and draw the undecided middle to their side, they attribute all deficits and social problems to the open, cosmopolitan-oriented society. They see pluralism as the root of all evil and oppose internationalism and supranationalism, immigration and any restriction of national sovereignty. Clearly, they advocate an end to the European consensus and promote aggressive migration policies and economic protectionism. Exclusive communitarian, nationalist ideas are combined with racism, antisemitism and islamophobia. In their European policy, these groups are undermining the European consensus. Nationalist and anti-European actors try to do this in three different ways. Leaving the EU is the explicit path that the British have already taken. But since this does not have the support of clear majorities in many countries, the Eurosceptics are trying to renationalise the EU from within. They succeed partly by renegotiating treaties and partly by ignoring or blocking EU laws in the European council and the European Parliament.

The attempt to establish exclusive and closed societies with populations that are as homogeneous as possible—a development that by some is interpreted as a reaction to globalisation—is itself challenged by cosmopolitan attempts to supranationalise or even globalise democracy. Cosmopolitans demand a European republic in which states no longer play a decisive role [23]. Some want to largely detach democracy from its representative structures with direct democratic procedures to replace them. In this context, little attention is paid to the fact that socio-structural inequality also contributes to inequality in democratic procedures, since certain competences to assert one’s own interests are less pronounced among the poorly educated. They are less used to speaking publicly, presenting their arguments and forming networks.

In an increasingly aggressive environment and a battle for undecided voters in the middle, cosmopolitan forces, often seen as the winners of globalisation, promote the unconditional overcoming of the nation state with its traditional ties. Two dividing failures or problems are mixed into this concern, which is in itself normatively demanding and positive in terms of human rights or climate policy: (1) the ignorance of socio-economic conditions and their impact on lifestyles which I call lifestyle arrogance and (2) the underestimation of the democratic qualities of nation states, the transferability of which to larger entities is not conceivable without problems.

Some dogmatic cosmopolitans thus make the mistake of using moral appeals and a certain lifestyle arrogance to defame those who, for socio-economic reasons or because of their socialisation, hold different world views [43]. Those who are undecided, but express doubts about the overcoming of the nation state or the radical change of their lifestyles, are portrayed as backward, nationalistic and unreasonable. Lifestyles thus become even more politicised. Only those who behave according to certain universal and ecological goals are considered responsible democrats. From a sociological point of view, a kind of new bourgeoisie threatens the old middle class [52]. Here, it is important to stress that it is not the basic idea of cosmopolitanism that is exclusionary, but the way it is promoted by some loud voices in the political debate. Socio-economic conditions are ignored; opponents of one’s own worldview are regarded as antagonists. National interests are seen as hurdles for a “European common good”. The democratic achievements of nation states are no longer recognised. They are considered easily transferable to a supranational or even global level [41]. Here, it is noticeable that even in the better educated strata of the globalisation winners, certain democratic competences and experiences of democracy are lacking. The fact that dialogue and participatory rationality are central components of democracies is not anchored in this group. As a result, they fall into the trap of polarisation and respond to the pushers of the other side with their own exacerbation campaign. Everyday life and its banalities are politicised. Everything is understood as an expression of political attitudes. What someone eats, how and where people live or work, if they have a car or not, where and how they spend their holidays, what music they listen to—all this is evaluated, ranked and devalued. In this scenario, some of the self-declared promoters of progressive cosmopolitanism divide the world into those with a sustainable or imperial lifestyle [8]. The private dissolves and becomes the object of steady evaluation in the political debate. In this irreconcilable positioning, political actors of the centre fail. Neither local nor regional, national or supranational authorities are able to build bridges. The split is heading towards a violent conflict, at the end of which one of the two groups will win at the expense of democracy.

Merkel and Zürn [42] have described the new cleavage in detail and shown how parties align and profile themselves along these questions. Their critique is less of the differentiated theoretical models of communitarianism and cosmopolitanism than of their political instrumentalisation. They see representational deficits of modern democracies as the source of the conflict. Institutions of democracy have no place for the so-called losers of globalisation. In this irreconcilable positioning, political actors of the centre fail. Neither local nor regional, national or supranational authorities are able to build bridges. Inequalities are widening. The places of dialogue and experience of democracy in everyday life remain rare. The split is heading towards a violent conflict, at the end of which one of the two groups will win at the expense of democracy.

### Scenario 2: Democratised Europe—relative cosmopolitanism and dialogue

In the second scenario, the initial situation is similar. Polarisers are driving society apart. Authoritarian populists with nationalist arguments face dogmatic cosmopolitans who sometimes cultivate a kind of lifestyle arrogance. The difference, however, arises in the response to the polarisation process. While in the first scenario there are no bridge-builders in a powerful position, in the second scenario a positive, democracy-promoting form of confrontation develops, based on a relative, rebellious cosmopolitanism. The reaction to the burgeoning exclusive and nationalist communitarianism is similar among some actors here as well. We assume here too, that some argue with moral appeals and disregard the realities of people’s lives. In contrast to the first scenario, however, relative cosmopolitans enter the scene and build bridges. To build democratic bridges, it is not the pushers who need to be addressed, but the undecided middle. Relative cosmopolitans avoid the two mistakes described in the first scenario. They are neither ignorant towards socio-economic and political inequality, nor are they naïve in terms of the transferability of democracy and the role of the nation state.

This cosmopolitism is relative also in the way as it does not regard the goal as an absolute dogma for which all means are justified and because it allows individuals and societies more differentiated paths to the goal. It is thus a cosmopolitanism that arises from the reality of life. It is rebellious because it includes resistance to every form of authoritarianism and to every kind of polarisation or division, not only on a political level, but on a daily life level. Dialogue is the basis of this relative, rebellious cosmopolitanism. It is what Albert Camus describes as the experience of someone who develops his solidarity from the experiences of poverty in a concrete situation [12]. It is thus not an academic or abstract universalism of a winner of globalisation, but the human solidarity of a person who develops cosmopolitanism solidarity in the experience of everyday life challenges. This turns cosmopolitanism to its humanist core. It is not founded on economic or intellectual superiority, not on the experience of being a winner, but on the contrary, on the experience of existential suffering. This can also be called cosmopolitanism from below or rooted cosmopolitanism [2, 4]. The crucial point is the one that Patrick Hayden emphasises with recourse to Camus: A cosmopolitanism that is not dogmatic, that does not itself become an ideology [28]. Michael F. Mascolo describes it from a psychological perspective and suggests a relational-dialectical approach for constructive political discourse. This approach builds bridges through dialectical engagement [37]. The inclusion and understanding of the other plays a central role in this. The characteristics of polarisation are mitigated by these strategies. Political opponents are not considered enemies. Instead of tugging at the undecided, different positions are allowed and discussed. There are a number of examples in history where such strategies based on dialogue achieved a positive effect (see [51, 57]).

In Scenario 2, the polarising strategies of the pushers are thus contrasted with a dialectical method of discourse, dialogue and democracy. However, in order to achieve this on a large scale, efforts are needed from different actors at different political levels, from the European Commission, the European Parliament, the ministries in the nation states, from regional and local authorities and finally from further organisations and interest groups. Two preconditions are necessary. At the level of legislation, a further increase in social inequality is prevented in this scenario. Rather, social action is taken to end poverty and promote inclusion. The risks of globalisation are no longer passed on to the individual, but are assumed by the state. At the same time, access opportunities to education, jobs and political participation are increased. The second crucial step is to improve the quality of democracy and dialogue. Efforts are needed here from the local level up to the supranational level. The end of the European consensus, which has long held as a permissive consensus, is being replaced by a discursive, public debate, the strengthening of the European Parliament and the introduction of different forms of participatory democracy [26].

Nation states are not replaced or overcome in this scenario, but seen as an example of how democracy can work, how dialogue and social partnership can be organised. Although they lose their veto-power, they are still crucial in decision making on the EU level as a kind of second chamber. The power of governments is not being replaced, but complemented by the power of citizens, through the further enhancement of the European Parliament and new forms of participation such as citizens’ councils at EU level [16]. However, this strengthening of the supranational level is only possible with the consent and involvement of the trade unions so that the so called losers of globalisation do not have to fear a further weakening of their interests, but quite the opposite [21]. For this to succeed, social dialogue is strengthened.

At national level, aspects of political equality are strengthened: transparency, representation and participation in parliaments and other institutions of representative democracy are actively promoted [34]. However, this is not happening because of new sanity or goodwill of the political parties, but because of public pressure arising from failures and corruption scandals. The nepotism that surfaces in many states draws public outrage and reform. These reforms are driven by examples where party control and citizen participation succeed. The citizens' councils and assemblies in Ireland, Belgium, French cities, etc. [53] inspire other states and show that democratic innovations do not have to remain elitist games, but that through certain and constantly improved procedures, less privileged groups can also gain access to political decision making. Strengthening citizenship education and democratic competences, as defined by the Council of Europe, in schools and educational institutions also plays an important role [32].

An attempt is made to create an ideal speech situation as described by Habermas in different contexts. It means that all participants have the same chance of initiation and participation in the debate. They evaluate each other’s assertion on the basis of reason and evidence and have an interest in rational consensus. The point is to exclude coercion from the debate as far as possible [24]. Even if there are justified doubts about this theory of communicative action with regard to its practicability and its many presuppositions, it can be considered an ideal to approach. Structural and social hierarchies shall be limited to a minimum. Especially at the local level, it is possible to improve the dialogue between the different population groups and their different interests [14].

In this scenario, already existing structures are expanded, promoted and funded by national and supranational authorities. This leads to people having experiences of democracy in their immediate environment that they would otherwise not have known. Bridge builders are strengthened in their work. Social workers ensure that their clients are empowered in their democratic competences. In this way, sections of the population that are most exposed to the risks of globalisation can bring their interests to bear, and it does not stop at articulation, but also leads to visible consequences. This takes the wind out of the pushers’ sails. Although they remain an important factor in the democratic game, they can no longer have the impact they had in scenario 1. The bridge builders, on the other hand, become stronger and more, motivated and encouraged by state institutions and laws that put an end to dangerous inequality.

Polarisation will not disappear in this scenario either, but it will be turned in a direction that is less dangerous and includes the chance of democratisation. In all measures, the concept of a relative, rebellious cosmopolitanism is at the forefront, which is not dogmatic and attempts to dialectically overcome the split between communitarian and cosmopolitan positions through dialogue and inclusion. Without question, this scenario is very demanding and therefore, at first glance, extremely unlikely. Above all, it cannot be assumed that all the measures mentioned will take place at all political levels at the same time. Nevertheless, already existing initiatives can certainly be strengthened by various actors. In the public debate, there is little knowledge about successful democratic innovations of different countries, regions or cities. If they receive more attention, this scenario is also less absurd than it might seem at first glance.

## Conclusion

The democratic and European consensus can no longer be taken for granted. Conflicts that hardly played a role or were covered up by a permissive consensus are now emerging in the form of a strong polarisation. How this situation can develop further was described in the article in two scenarios. In scenario 1 “Polarised Europe”, polarisation is driven by authoritarian populists and anti-democratic extremists, but also by those who, through moralising, elevate their own position vis-à-vis all others. This leads to a hardening of positions between exclusive communitarians and dogmatic cosmopolitans. The situation continues to escalate. At the political level, this makes negotiation between parties and thus the formation of governments in different states ever more difficult. The division is also growing in the broader population. Political opponents are increasingly seen as enemies with whom discussion is avoided. The end of dialogue increases the risk of violence and mutual exclusion. The quality of democracy suffers from this development. Equal political freedom, human and citizens’ rights, dialogue and solidarity between citizens are weakened. Government formations take longer. Parliamentary debates are taken less seriously or are ridiculed, while the confrontation in the streets increases. People think in categories of friend and enemy, as Carl Schmitt described it. Europe is strongly polarised and does not find solutions for the ever growing conflicts. Representative democracy and the European integration process are questioned and social cohesion is sustainably weakened.

In the second scenario “Democratised Europe”, the model of relative cosmopolitanism pays attention to the realities of life of all groups without moralising and offers solutions through dialogue. Relative cosmopolitanism, inspired by Albert Camus and described in detail by Patrick Hayden [28], assumes the equality of all people and is based on a humanistic worldview, but recognises that this very worldview does not emerge of its own accord but depends on democratic experiences of citizens, structural and economic realities, political socialisation and citizenship education. The attempt to enable equal access to political decision-making is in the foreground. The inclusion of the other [25] is made possible through dialogue. Relative cosmopolitanism is not only represented by an educated elite, but also by those who live a kind of cosmopolitanism from below in a globalised world. However, it is not just a matter of improving dialogue forums and democratic innovations. In the second scenario, socio-economic inequalities and the marginalisation or exclusion of certain groups are also addressed. Polarisation is mitigated and constructively turned into a more inclusive and democratic Europe.

## Data Availability

Data sharing is not applicable to this article as no datasets were generated or analysed during the current study.
